# Sex-Specific Transcriptomic Signatures in Brain Regions Critical for Neuropathic Pain-Induced Depression

**DOI:** 10.3389/fnmol.2022.886916

**Published:** 2022-05-18

**Authors:** Weiping Dai, Shuying Huang, Yuan Luo, Xin Cheng, Pei Xia, Mengqian Yang, Panwu Zhao, Yingying Zhang, Wei-Jye Lin, Xiaojing Ye

**Affiliations:** ^1^Faculty of Forensic Medicine, Zhongshan School of Medicine, Sun Yat-sen University, Guangzhou, China; ^2^Guangdong Province Translational Forensic Medicine Engineering Technology Research Center, Sun Yat-sen University, Guangzhou, China; ^3^Guangdong Province Key Laboratory of Brain Function and Disease, Zhongshan School of Medicine, Sun Yat-sen University, Guangzhou, China; ^4^Guangdong Provincial Key Laboratory of Malignant Tumor Epigenetics and Gene Regulation, Guangdong-Hong Kong Joint Laboratory for RNA Medicine, Medical Research Center, Sun Yat-sen Memorial Hospital, Sun Yat-sen University, Guangzhou, China

**Keywords:** neuropathic pain, sex-differences, depression, medial prefrontal cortex, anterior cingulate cortex, RNA sequencing

## Abstract

Neuropathic pain is a chronic debilitating condition with a high comorbidity with depression. Clinical reports and animal studies have suggested that both the medial prefrontal cortex (mPFC) and the anterior cingulate cortex (ACC) are critically implicated in regulating the affective symptoms of neuropathic pain. Neuropathic pain induces differential long-term structural, functional, and biochemical changes in both regions, which are thought to be regulated by multiple waves of gene transcription. However, the differences in the transcriptomic profiles changed by neuropathic pain between these regions are largely unknown. Furthermore, women are more susceptible to pain and depression than men. The molecular mechanisms underlying this sexual dimorphism remain to be explored. Here, we performed RNA sequencing and analyzed the transcriptomic profiles of the mPFC and ACC of female and male mice at 2 weeks after spared nerve injury (SNI), an early time point when the mice began to show mild depressive symptoms. Our results showed that the SNI-induced transcriptomic changes in female and male mice were largely distinct. Interestingly, the female mice exhibited more robust transcriptomic changes in the ACC than male, whereas the opposite pattern occurred in the mPFC. Cell type enrichment analyses revealed that the differentially expressed genes involved genes enriched in neurons, various types of glia and endothelial cells. We further performed gene set enrichment analysis (GSEA), which revealed significant de-enrichment of myelin sheath development in both female and male mPFC after SNI. In the female ACC, gene sets for synaptic organization were enriched, and gene sets for extracellular matrix were de-enriched after SNI, while such signatures were absent in male ACC. Collectively, these findings revealed region-specific and sexual dimorphism at the transcriptional levels induced by neuropathic pain, and provided novel therapeutic targets for chronic pain and its associated affective disorders.

## Introduction

Neuropathic pain, which is defined as “pain caused by a lesion or disease of the somatosensory system,” affects 6–8% of the population worldwide (Treede et al., 2008; Bannister et al., 2020; Finnerup et al., 2021). As a debilitating medical condition, neuropathic pain severely impairs the quality of life of patients due to the difficulty in clinical management of the disease (Treede et al., 2008). Over 60% of patients with neuropathic pain have depression, which is characterized by persistent depressed mood as well as loss in pleasure and motivation (Bair et al., 2003; de Heer et al., 2014). Such high comorbidity with depression in patients suffering from neuropathic pain exacerbates the severity of pain sensation and leads to worsening prognosis (Williams et al., 2003; Walker et al., 2014; Tappe-Theodor and Kuner, 2019). However, the mechanisms underlying the dynamic interactions of neuropathic pain and depression remain poorly understood. Moreover, although pain and depression can occur in both sexes, women generally show higher prevalence of physical pain and depression than men, while the underlying mechanisms remain underexplored (Ruau et al., 2012; Altemus et al., 2014; Vetvik and MacGregor, 2017; Mogil, 2020; Bangasser and Cuarenta, 2021).

The experience of pain, a combination of sensory and emotional components, is thought to arise from collaborative activities of multiple brain regions (Bushnell et al., 2013; Tan and Kuner, 2021). The brain regions most commonly activated by painful stimuli are often referred to as the “pain-related regions.” The medial prefrontal cortex (mPFC) and the anterior cingulate cortex (ACC) are considered as two hub regions (Tracey and Johns, 2010; Ong et al., 2019; Kummer et al., 2020). Both the mPFC and the ACC are associative cortices, with vast connectivity with other cortical and subcortical regions involved in the processing of pain and/or emotion (Etkin et al., 2011; Ong et al., 2019; Tan and Kuner, 2021; Pizzagalli and Roberts, 2022). These two regions are close in location, extensively connected with each other, and both can be activated by various types of acute pain (Bliss et al., 2016; Seminowicz and Moayedi, 2017; Tan and Kuner, 2021).

Chronic pain, including neuropathic pain, modifies the structures and functions of the mPFC and the ACC differently. Both human and animal studies have shown that chronic pain reduces the volume of gray matter as well as the activity of mPFC (Rodriguez-Raecke et al., 2009; Bushnell et al., 2013; Fritz et al., 2016; Phelps et al., 2021). The reduced mPFC activity is accompanied by increased GABAergic tone and decreased glutamatergic currents (Zhang et al., 2015; Kelly et al., 2016; Kummer et al., 2020). Pharmacologic or optogenetic rescue of mPFC activity alleviates nociceptive responses as well as pain-associated depression, suggesting that both the sensory and affective aspects of pain can be processed by the mPFC (Millecamps et al., 2007; Zhang et al., 2015; Hare and Duman, 2020; Tan and Kuner, 2021; Yan and Rein, 2021). Chronic pain also results in reduced gray matter volume in the ACC (Rodriguez-Raecke et al., 2009; Bushnell et al., 2013). However, a hyperactive phenotype of ACC was observed in human functional magnetic resonance imaging (fMRI) studies and confirmed by electrophysiological recording in the animal models of chronic pain (Buffington et al., 2005; Bliss et al., 2016). Prolonged pain induces long-term changes in synaptic plasticity, leading to persistently increased excitatory and decreased inhibitory synaptic transmission in the ACC (Li et al., 2010; Bliss et al., 2016). Lesion or inactivation of ACC relieves neuropathic pain-induced depression, but the effects on nociception are mixed (Barthas et al., 2015; Tan and Kuner, 2021; Zhu et al., 2021). In the reversed fashion, electrical or optogenetic activation of ACC lowers mechanical pain threshold as well as induces aversion to the place where it is delivered (Navratilova and Porreca, 2014; Bliss et al., 2016; Zhang et al., 2017; Tan and Kuner, 2021). Therefore, in contrast to the impairment of the mPFC, chronic pain induces abnormal elevation of the ACC activity to increase pain sensitivity and drive depression. However, the differential mechanisms underlying acute pain-induced activation of both the mPFC and the ACC, and the opposite changes induced by prolonged pain in these two brain regions, remain to be explored.

The comorbidity of neuropathic pain and depression engages long-lasting functional and structural modifications of the mPFC and the ACC, which in turn often involve changes in gene expression that are functionally associated with both diseases (Bliss et al., 2016; Descalzi et al., 2017; Li et al., 2021; Tan and Kuner, 2021). In this study, we aimed to compare differences in the transcriptomic profiles of the mPFC and the ACC in both female and male mice induced by spared nerve injury (SNI), a classical model of neuropathic pain (Decosterd and Woolf, 2000; Guida et al., 2020; Sadler et al., 2022). Our results revealed that more robust transcriptomic changes were induced at 2 weeks after SNI in the ACC of female mice than that of male mice, whereas the opposite pattern was observed in the mPFC. Further analyses revealed brain region-specific and sexual dimorphic changes in the transcriptional profiles after SNI. These findings are of value to advance the understanding of the mechanisms underlying cortical processing of pain and its affective aspects, and to suggest novel therapeutic targets for the treatment of neuropathic pain and its associated affective disorders.

## Materials and Methods

### Animals

A total of 2–3 months old female and male C57BL/6J mice were obtained from the Institute of Experimental Animals of Sun Yat-sen University or Guangdong Medical Laboratory Animal Center (Guangzhou, China). The mice were housed in groups of 5 and maintained on a 12 h light/dark cycle in a specific pathogen free (SPF) facility. All mice were allowed *ad libitum* access to food and water. The experiments were performed during the light cycle. The animals were randomly assigned to different experimental groups and were handled for 2 min per day for 4 days before behavioral testing. All animal studies were approved by the Institutional Animal Care and Use Committee of Sun Yat-sen University. A total number of 132 animals were used in the study.

### Spared Nerve Injury Surgery

Mice were anesthetized with 1.5% isoflurane at an oxygen flow rate of 0.4 L/min (RWD Life Science, Shenzhen, China). After shaving the fur and sterilizing the incision site, incisions were made in the skin and muscle on the left hind leg at the mid-thigh level to expose the sciatic nerve and its branches. The common peroneal and sural nerves were ligated with 5.0 silk suture (Shanghai Pudong Jinhuan Medical Instrument, Shanghai, China), transected, and a 1–2 mm sections of each nerve were removed. The tibial nerve was left intact. Afterward, the skin was sutured. The mice were allowed to recover on a heating pad before returning to their home-cage. The control mice were sham operated, exposed to the same surgery procedure without injury of the nerves.

### von Frey Test

The sensitivity to mechanical pain threshold was measured following the up-down method using the von Frey hairs (Aesthesio Precise Tactile Sensory Evaluator Kit, DanMic Global Danmic, United States) (Hansson, 2003; Deuis et al., 2017). Mice were habituated to a transparent chamber with a metal mesh floor for 30–60 min before testing. The plantar surface of the hind paw was stimulated with von Frey hairs and a quick withdrawal of the paw upon the stimulation was indicative of a nociceptive response. von Frey hairs with forces ranging from 0.04 to 2 g were applied in an ascending manner, and each hair was applied for five consecutive times. The lowest force to evoke a paw withdrawal 50% of the time was recorded as the threshold for mechanical pain (Chaplan et al., 1994). von Frey test was performed by an investigator who was blinded to the experimental conditions.

### Behavioral Tests

Depression-like behaviors were assessed over 2 and 4 weeks after the surgery of SNI, as previously described (Cryan et al., 2005; Jiang et al., 2019; Vuralli et al., 2019). The animals were acclimated to the testing room for 1–2 h prior to testing. The behavioral testing apparatus were cleaned with 75% ethanol between animals. Behavioral analyses were performed by an investigator who was blinded to the experimental conditions.

The splash test (ST) was carried out in a normal mouse home-cage under red-light. The mouse was allowed to habituate in the cage for 1 min, after which the dorsal coat of mouse was sprayed with a 10% sucrose solution. The test process was videotaped, and the grooming time in the first 5 min after the application of sucrose solution was scored by a researcher blinded to the experimental groups.

The tail suspension test (TST) was carried out by suspending the individual mouse by its tail from a ledge with an adhesive tape (approximately 1 cm from the tip of the tail). The immobile time during the 5 min of the test were videotaped and analyzed offline by a researcher blinded to the experimental groups. The mice were considered immobile only when they were hung passively and completely motionless.

The forced swim test (FST) was carried out by placing the mice into a 5 L glass beaker containing 3.5 L of water (24–25°C) under white light, and videotaped for 6 min. The water in the cylinder was replaced after each animal. The immobile time during the last 4 min of the 6 min test were analyzed offline by a researcher blinded to the experimental groups. The mice were defined as immobile as absence of any movement except that necessary for them to keep their heads above water.

The open field test (OFT) was used to assess the general locomotor activity of the mice. The mice were placed onto a corner of a 40 × 40 cm arena illuminated with red-light. The total distance traveled in the 5-min test were tracked and analyzed by the DigBehv system (Ji-Liang, Shanghai, China).

### Tissue Dissection and RNA Extraction and Sequencing

Two weeks after SNI surgery or sham operation, mice were sacrificed by rapid cervical dislocation. Their brains were sliced into 1 mm sections on a brain matrix (RWD Life Science, Shenzhen, China) in ice-cold dissection buffer (2.6 mM KCl, 1.23 mM NaH_2_PO_4_, 26.2 mM NaHCO_3_, 5 mM kynurenic acid, 212.7 mM sucrose, 10 mM dextrose, 0.5 mM CaCl_2_, 1 mM MgCl_2_). The mPFC and ACC were dissected out with a 15G puncher and snap-frozen on dry ice. Total RNA was isolated using RNAeasy Micro Kit following the manufacturer’s protocol (Qiagen, Hilden, Germany). The RNA samples were submitted to the MAGIGENE (Guangzhou, China) for quality control using Agilent 4200 Bioanalyzer. All samples have RNA integrity numbers (RINs) > 8. The samples further underwent library construction and sequenced by an Illumina NovaSeq 6000 system as paired-end 150 bp reads.

### RNA Sequencing Alignment, Read Counting and Differential Gene Expression Analysis

Raw data of fastq format was processed by Trimmomatic (version 0.36) to acquire the clean reads, which were then mapped to NCBI Rfam databases, to remove the rRNA sequences by Bowtie2 (version 2.33). The reads were mapped to the mouse reference genome^1^ using the Hisat2 (version 2.1.0) (Anders et al., 2015; Lachmann et al., 2020). HTSeq-count (version 0.9.1) was used to obtain the read count and function information of each gene. The count tables were normalized based on their library size using trimmed mean of *M*-values (TMM) normalization implemented in R/Bioconductor EdgeR (version 3.34.0) (Robinson et al., 2010; McDermaid et al., 2019). Normalized read counts were fitted to a negative binomial distribution with a quasilikelihood *F*-test. Principal component analysis (PCA) was performed for the regularized log transform (rlog) of the normalized counts using plotPCA tools with default parameters (Mi et al., 2019; Yoo et al., 2021). Differential gene expression analysis was further carried out using EdgeR. The transcripts were considered as differentially expressed genes (DEGs) at false discovery rate (FDR) < 0.1 with Benjamini–Hochberg correction for multiple testing. Volcano plots, Venn plot and heatmaps were generated using VennDiagram (version 1.6.20), plot, and pheatmap (version 1.0.12) packages in R/Bioconductor (Wang et al., 2021; Yoo et al., 2021).

### Cell Type-Specificity Analyses

To identify cell type-enriched transcripts, we compared our DEGs to a database of cell type-specific mRNA expression published by Zhang et al. (2014), which established selectively enriched transcripts in neurons, glia, and vascular cells of mouse cerebral cortex. Using FPKM numbers for astrocyte, endothelial cell, neuron, microglia, and oligodendrocyte, we calculated the enrichment scores of the transcripts as follows: enrichment score in cell type X = FPKM of transcripts expressed in cell type X/FPKM of transcripts expressed in all other cell types. The DEGs with enrichment scores >1.5 in a given cell type were considered as cell-type enriched.

### Validation of RNA Sequencing Data by Quantitative PCR

The quantitative PCR (qPCR) primers were selected from the PrimerBank^2^ or designed using Primer-BLAST.^3^ The specificity of the primers was further confirmed with BLAST^4^ and melting curve analysis, and the amplification efficient of the primers were examined by qPCR using series dilutions of a cDNA template. The sequences of the primers are listed in Supplementary Table 1. The cDNAs were synthesized using the NovoScript Plus All-in-on Strand cDNA Synthesis Supermix (Novoprotein, Suzhou, China) following the manufacturer’s instructions. The qPCR was performed using a CFX96 Touch Real-Time PCR Detection System (Bio-Rad) with the NovoStart SYBR qPCR SuperMix Plus (Novoprotein, Suzhou, China). For each sample, 2 or 8 ng of cDNA was amplified using one initial denaturation step at 95°C for 1 min, followed by 40 cycles at 95°C for 20 s, 60°C for 20 s, and 72°C for 30 s. Triplicates of each sample were analyzed by qPCR, and the mean cycle quantification (Cq) value was used for calculating the relative expression of target mRNAs using the ΔΔCt method, using the mRNA level of *Gapdh* as the internal control for normalization.

### Analyses of Protein–Protein Interaction Networks

STRING (version 11.5) was used to identify potential protein–protein interactions between the DEGs. The cytoHubba’s Maximal Clique Centrality (MCC) score were used to identify top hub DEGs and their sub-networks (Chin et al., 2014; Szklarczyk et al., 2019; Pan et al., 2021). The data were visualized by Cytoscape (version 3.9.1).

### Gene Set Enrichment Analysis

Gene set enrichment analysis (GSEA) (Broad Institute, version 4.2.2) analysis was performed to identify changes in functional enrichments of the transcriptomic profiles, using the gene set databases for Gene Ontology (GO, c5.go.bp.v7.51.symbols.gmt, c5.go.mf.v7.51.symbols.gmt, and c5.go.cc.v7.51.symbols.gmt) and KEGG pathways (c2.cp.kegg.v7.51.symbols.gmt). Gene set size filters were set at minimum of 5 and maximum of 1000. FDR for the enrichment score of the gene set were calculated based on 1000 gene set permutations. The top gene sets enriched in each group were plotted with ggplot2 (version 3.3.5) in R.

### Statistical Analyses

The statistical analyses of the RNA sequencing (RNAseq) data were described in the sections above. Two-way ANOVA followed by Sidak’s or Tukey’s multiple comparisons tests were used for comparing the behavioral or von Frey test results for the sham versus SNI groups of female and male mice. Unpaired Student’s *t*-test was used for qPCR analyses to validate gene expression changes by SNI. The statistical analyses were performed in GraphPad Prism for windows (version 8.0.0). Data were presented as mean ± SEM and *p* < 0.05 was considered as statistically significant.

## Results

### Spared Nerve Injury Induces Depression-Like Behaviors in Both Female and Male Mice

**FIGURE 1:**
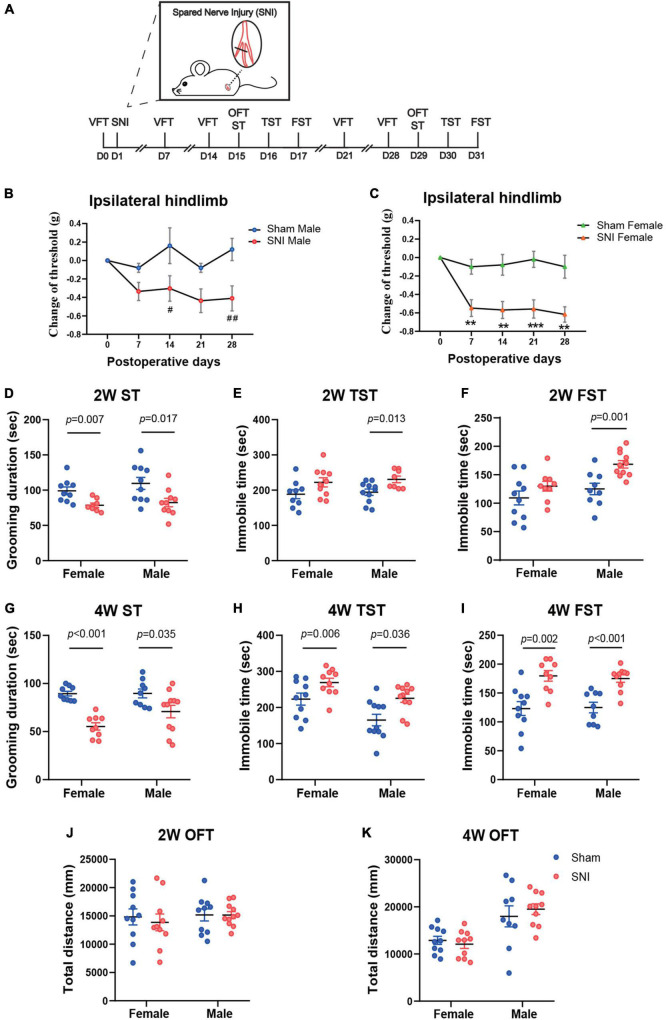
The spared nerve injury (SNI) model of neuropathic pain induces mechanical allodynia and depression-like behaviors in both female and male mice at 2 and 4 weeks after surgery. **(A)** The schema for SNI and the experimental timeline showing the sequence of surgery and behavioral tests. **(B,C)** Changes in the mechanical withdrawal threshold of the ipsilateral hindlimb over 4 weeks after surgery, compared to the threshold measured before surgery by von Frey test (VFT). Data are presented as mean ± SEM and analyzed by two-way ANOVA followed by Tukey’s multiple comparisons test. ^#^*p* < 0.05, ^##^*p* < 0.01 for SNI male group versus Sham male group **(B)**; **p < 0.01, ***p < 0.001 for SNI female group versus Sham female group **(C)**. *N* = 10 (sham female), 10 (SNI female), 5 (Sham male), and 11 (SNI male). The time spent in grooming during the 5 min splash test (ST) at 2 weeks **(D)** and 4 weeks **(G)** after SNI surgery. The time spent immobile during the 5 min tail suspension test (TST) at 2 weeks **(E)** and 4 weeks **(H)** after SNI surgery. The time spent immobile during the last 4 min of the 6 min forced swim test (FST) at 2 weeks **(F)** and 4 weeks **(I)** after SNI surgery. The total distance traveled during the open field test (OFT) at 2 weeks **(J)** and 4 weeks **(K)** after SNI surgery. **(D–K)** *N* = 9–11 mice per group. Data are analyzed by two-way ANOVA followed by Sidak’s multiple comparisons test, and presented as mean ± SEM.

To explore the temporal course for the development of depressive symptoms after SNI, a classic model of neuropathic pain, we measured behavioral changes at different time points after SNI surgery in both female and male mice. The von Frey test was used to measure mechanical pain sensitivity. The ST, TST, and the FST were used to assess depressive-like behaviors. The OFT was used to evaluate general locomotion (Figure 1A). The results showed that, after SNI, mice of both sexes developed robust mechanical allodynia as early as 1 week post-surgery, which persisted for at least 4 weeks. The decrease in mechanical pain threshold measured by the von Frey test was relatively larger in the female mice than that in the male mice (Figures 1B,C). At 2 weeks after SNI surgery, the female mice exhibited significantly less grooming time in the ST and trends toward increased immobility in the TST and the FST. Similarly, the male mice also showed depressive phenotypes at 2 weeks post-SNI surgery, including significantly decreased grooming time in the ST and increased immobility in the TST and FST (Figures 1D–F). At 4 weeks after SNI surgery, both female and male mice showed robust depression-like behaviors (Figures 1G–I). The decreased grooming and increased immobility in the tests above were unlikely due to the impairment in the general locomotion of these mice after SNI surgery, as for both time points, the total distance of mice traveled in the OFT was not altered (Figures 1J,K).

Thus, these data reveal that SNI induces chronic pain and depression-like behaviors in both female and male mice.

### Sex-Specific Transcriptional Signatures in the Medial Prefrontal Cortex and the Anterior Cingulate Cortex of Mice After Spared Nerve Injury Surgery

We next sought to identify transcriptional changes that may contribute to the development of depressive symptoms induced by neuropathic pain. Previous studies have reported transcriptomic changes in the brains after SNI but at much later time points (2.5–6 months post-surgery) (Alvarado et al., 2013; Berta et al., 2017; Descalzi et al., 2017; Li et al., 2021). Since the mPFC and the ACC are two brain regions critical for regulating both the affective symptoms of neuropathic pain and depression (Bliss et al., 2016; Descalzi et al., 2017; Li et al., 2021; Tan and Kuner, 2021), we chose to examine the transcriptomic profiles of the mPFC and the ACC at 2 weeks post-SNI, a time point when the mice started to show mild depressive phenotypes, to identify the potential molecular signatures that are involved in the emergence of depressive phenotypes during the progression of neuropathic pain.

**FIGURE 2:**
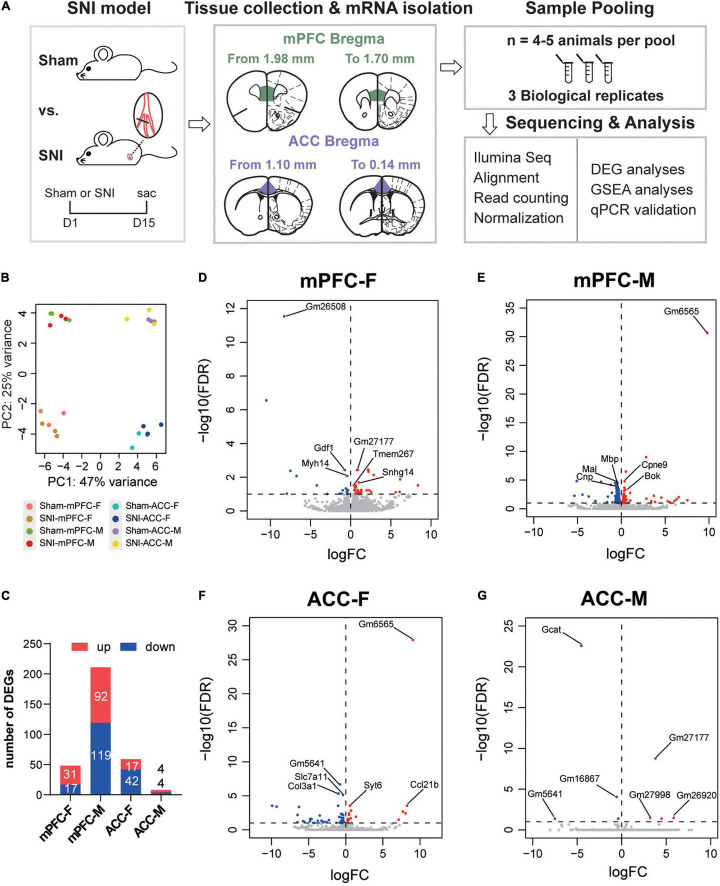
Differentially expressed genes in the mPFC and the ACC of female and male mice at 2 weeks after SNI surgery, compared to the sham group. **(A)** Experimental design and the workflow of the RNAseq and analyses. **(B)** Principal component analysis (PCA) plot of the sham versus the SNI groups, depicting the clustering of samples: the mPFC versus the ACC, female versus male, and the sham group versus the SNI group. **(C)** The numbers of the significantly upregulated or downregulated differentially expressed genes (DEGs) by SNI. mPFC-F, the mPFC of female mice; mPFC-M, the mPFC of male mice; ACC-F, the ACC of female mice; ACC-M, the ACC of male mice. The volcano plots of the DEGs in the mPFC of female (mPFC-F, **D**) and male mice (mPFC-M, **E**), as well as in the ACC of female (ACC-F, **F**) and male mice (ACC-M, **G**), with horizontal lines at the –log_10_ (false discovery rate, FDR) = 1 and vertical lines at log_2_ (fold change, FC) = 0, delineating positive versus negative regulation. Blue dots represent downregulated DEGs, and red dots represent upregulated DEGs.

The mPFC and the ACC were harvested from female and male mice at 2 weeks after SNI surgery or sham operation. The RNAs from both groups were extracted in parallel and underwent quality checks. Afterward, RNAseq were performed on three independent pools of samples from each sex and brain region (4–5 animals were mixed as one biological pool) (Figure 2A). The principal component analysis (PCA) revealed striking separation between the mPFC and the ACC transcriptomes, as well as between the female and male transcriptomes (Figure 2B). In general, more differentially expressed genes between the sham and the SNI groups (DEGs, FDR < 0.1 by edgeR) were observed in the mPFC than the ACC (Figure 2C and Supplementary Table 2). Interestingly, for the mPFC, more DEGs were identified in the male mice (211 total) than the female mice (48 total). In contrast, for the ACC, more DEGs were identified in the female mice (59 total), whereas only 8 DEGs were identified in the male mice (Figure 2C).

Of note, among the top transcripts upregulated by SNI in the mPFC of SNI female mice, *Snhg14* encodes a long non-coding RNA that regulates the expression of ubiquitin protein ligase E3A (*Ube3a*), which is known to critically implicate in the social interaction dysfunction in Angelman’s syndrome (Stanurova et al., 2016; Chung et al., 2020). The top transcripts downregulated in the mPFC of SNI female mice involved *Gdf1*, which encodes growth differentiation factor 1, a secreted ligand of the TGF-beta superfamily (Tanaka et al., 2007), and *Myh14*, which encodes the heavy chain of myosin, a motor protein (Donaudy et al., 2004; Figure 2D). For the mPFC of SNI male mice, the most noticeable signatures included downregulation of *Mal* (myelin and lymphocyte protein), *Mbp* (myelin basic protein), and *Cnp* (2′,3′-cyclic nucleotide 3′-phosphodiesterase), genes that are known for their roles in myelin formation (Fulton et al., 2010; Cao et al., 2013; Figure 2E). The noteworthy transcriptomic changes in the ACC of female mice included upregulation of *Syt6* and *Syt2*, which encode presynaptic protein synaptotagmins and are important for neurotransmitter release (Wolfes and Dean, 2020), and downregulation of *Col3a1* and *Col1a1*, two transcripts that encode type I and type III collagens, components of the extracellular matrix (Fujiwara et al., 2010; Santoro Belangero et al., 2018; Figure 2F and Supplementary Table 2). The eight DEGs identified in the ACC of male mice were mostly pseudogenes whose functions remain unclear, except *Gcat*, which encodes glycine C-acetyltransferase that is involved in the synthesis of glycine and acetyl-CoA (Ravichandran et al., 2018; Figure 2G).

**FIGURE 3:**
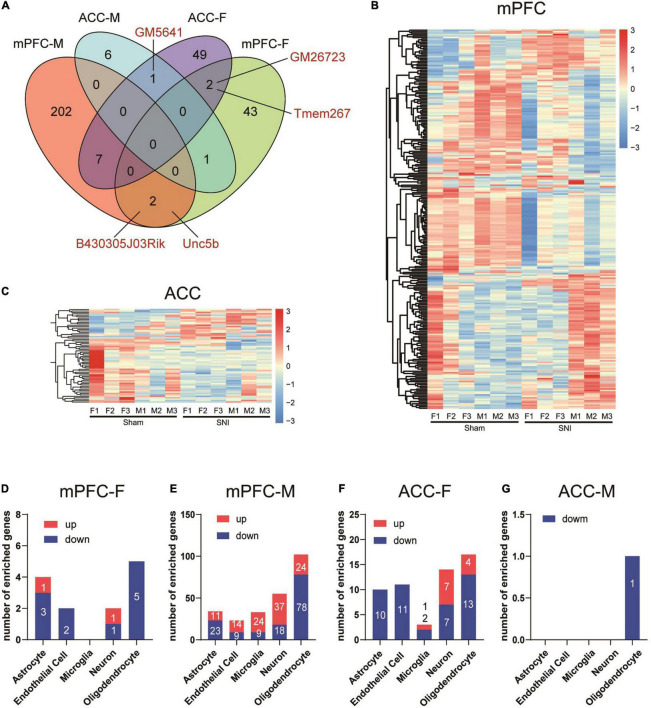
Differential transcriptomic profiles and cell-type enrichment of the DEGs induced in the mPFC and the ACC of mice of different sexes by SNI. **(A)** Venn diagram depicting limited overlaps of the DEGs between the mPFC and the ACC, as well as between different sexes. mPFC-F, the mPFC of female mice; mPFC-M, the mPFC of male mice; ACC-F, the ACC of female mice; ACC-M, the ACC of male mice. Hierarchical clustered heatmaps of the mPFC **(B)** and the ACC **(C)**, showing relative expression of the DEGs across the sham and the SNI groups in different sexes (F, female; M, male). Color scales represent standardized rlog-transformed values across samples. **(D–G)** The numbers of DEGs enriched in different cell types in the mPFC and the ACC of female and male mice, comparing the sham versus the SNI groups, as determined from a database of cell type-specific transcripts in the mouse cortex by Zhang et al. (2014).

Further comparison of the DEGs between different groups revealed little commonality between brain regions and between different sexes. In comparison of the mPFC and the ACC, we identified two common DEGs in the female mice (*Gm26723* and *Tmem267*) and no common DEGs in the male mice. By comparing female and male mice, we found two common DEGs in the mPFC (*B430305J03Rik* and *Unc5b*) and one common DEG in the ACC (*Gm5641*) (Figure 3A). We combined the DEGs from both sexes to generate the heatmaps, and the results revealed that part of the DEGs exhibited similar directions of changes in both sexes but with greater magnitudes in one sex over the other, while the other DEGs showed sex-specific changes and even changes in opposite directions in different sexes (Figures 3B,C).

We further evaluated the cell-type specificity of the identified DEGs by comparing our data to a cell-type specific mouse brain RNAseq repository (Zhang et al., 2014). For the DEGs in the female mPFC, 13 were identified to be enriched in specific brain cell-types, including astrocytes, endothelial cells, neuron and oligodendrocytes (Figure 3D). For the male mPFC, a noteworthy signature was downregulation of DEGs enriched in the oligodendrocytes (Figure 3E). For the DEGs in the ACC of female mice, the downregulated DEGs were found mainly enriched in astrocytes, endothelial cells, and oligodendrocytes, whereas the upregulated DEGs were found mainly enriched in neurons (Figure 3F). For the eight DEGs identified in the male ACC, one of those was enriched in oligodendrocytes (Figure 3G). Thus, SNI modifies the transcriptomic profiles across multiple cell-types in the mPFC and the ACC in both sexes.

**FIGURE 4:**
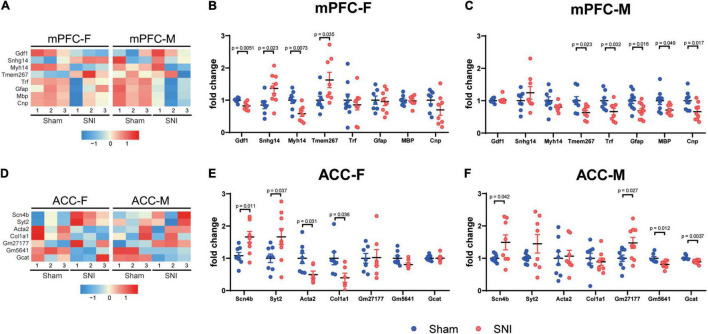
Quantitative PCR validation of the DEGs between the sham and the SNI groups in the mPFC and the ACC of mice of different sexes. Heatmaps showing relative expression levels of the selected upregulated and downregulated DEGs in the mPFC **(A)** and the ACC **(D)** of female and male mice, comparing the sham versus SNI groups. mPFC-F, the mPFC of female mice; mPFC-M, the mPFC of male mice; ACC-F, the ACC of female mice; ACC-M, the ACC of male mice. Color scales represent standardized rlog-transformed values across samples. qPCR validation results of the selected upregulated and downregulated transcripts in the mPFC **(B,C)** and ACC **(E,F)**. *N* = 6–9 mice per group. Data are analyzed by unpaired Student’s *t*-test, and presented as mean ± SEM.

To validate the RNAseq results, we performed qPCR analyses using independent sets of individual RNA samples. Eight mPFC-specific DEGs were chosen for the subsequent qPCR validation, which included *Gdf1* (growth differentiation factor 1), *Snhg14* (small nucleolar RNA host gene 14), *Myh14* (myosin heavy chain 14), *Tmem267* (transmembrane protein 267), *Trf* (transferrin), *Gfap* (glial fibrillary acidic protein), *Mbp* (myelin basic protein), and *Cnp* (2′,3′-cyclic nucleotide 3′ phosphodiesterase) (Figure 4A). Similarly, seven ACC-specific DEGs were assessed, which included *Scn4b* (sodium voltage-gated channel beta subunit 4), *Syt2* (synaptotagmin 2), *Acta2* (actin alpha 2), *Col1a1* (collagen type I alpha 1 chain), *Gm27177*, *Gm5641*, and *Gcat* (glycine C-acetyltransferase) (Figure 4D). The results of qPCR analysis confirmed the findings of the RNAseq (Figures 4B,C,E,F).

Collectively, our data showed that highly distinct and sex-specific changes in the transcriptomic profiles occurred in the mPFC and the ACC at 2 weeks after SNI surgery.

### Analysis of Protein–Protein Interaction Network Identifies Hub Genes Related to Spared Nerve Injury in the Male Medial Prefrontal Cortex and the Female Anterior Cingulate Cortex

**FIGURE 5:**
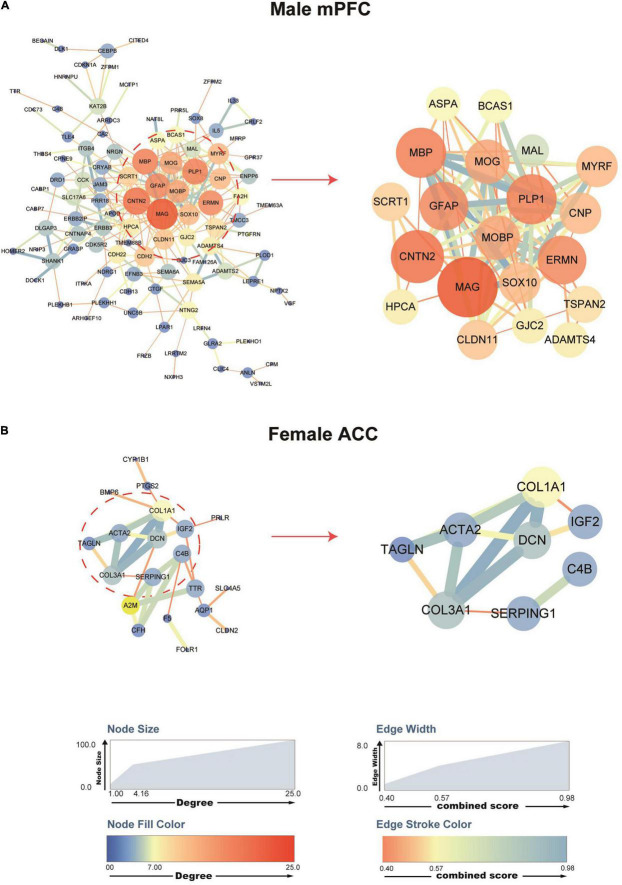
Protein–protein interaction (PPI) networks of the DEGs in the male mPFC and female ACC, comparing the sham versus the SNI groups. STRING PPI networks depicting the potential interactions of the DEGs in the mPFC **(A)** and the ACC **(B)** at 2 weeks after SNI. The color and size of the nodes are relative to the degree of the connectivity, and the color and size of the edges are relative to the combined score of the interactions.

To explore the molecular interactions and the potential key drivers of the identified DEGs in the mPFC and the ACC of female and male mice after SNI surgery, we performed network analysis based on the STRING Protein–Protein Interaction database (Szklarczyk et al., 2019). Analysis of the DEGs generated a network of 105 nodes and 249 edges in male mPFC, and 20 nodes and 30 edges in female ACC (Figures 5A,B). We did not observe any network structure for the female mPFC and the male ACC, likely due to the limited number of DEGs identified in these two conditions. We further used the cytoHubba’s MCC score to identify top hub DEGs and their sub-networks (Chin et al., 2014; Li and Xu, 2019). For the male mPFC, the top 20 hub DEGs constituted a sub-network, collectively engaged in nervous system development and axon ensheathment (Figure 5A). For the female ACC, the top eight hub DEGs formed a sub-network, encoding the components of extracellular matrix (Figure 5B).

Of note, among the top hub DEGs in the network for male mPFC, *Mal* (myelin and lymphocyte protein), *Cnp* (2′,3′-cyclic nucleotide 3′-phosphodiesterase), *Plp1* (proteolipid protein 1), *Mobp* (myelin associated oligodendrocyte basic protein), *Mbp* (myelin basic protein), and *Mog* (myelin oligodendrocyte glycoprotein) were functionally related to the myelination process and also among the top 20 DEGs whose expression was most significantly downregulated after SNI. Similarly, in the female ACC network, the hub DEGs, including *Col3a1* (collagen type III alpha 1 chain) and *Dcn* (decorin), were important components of the extracellular matrix and they were also among the top 20 DEGs whose expression was most significantly downregulated after SNI. These data suggest that decreased myelination in the male mPFC and disruption of extracellular matrix in the female ACC are likely the key factors involved in the pathological development of the comorbidity of SNI-induced neuropathic pain and depressive phenotype.

### Gene Set Enrichment Analysis of Spared Nerve Injury Versus Sham Groups Reveals Sex-Dependent Signatures in the Medial Prefrontal Cortex and the Anterior Cingulate Cortex

In order to gain functional insights of the transcriptomic changes in the mPFC and the ACC at 2 weeks after SNI surgery, we performed GSEA analysis, which does not rely on the DEGs selected by arbitrary imposed statistical cut-off parameters, but instead uses the entire list of genes ranked according to a combinational score of the fold change and the adjusted *p*-value. Thus, GSEA analysis is a sensitive method for GO/KEGG enrichment of genes with modest but coordinated changes (Subramanian et al., 2005).

**FIGURE 6:**
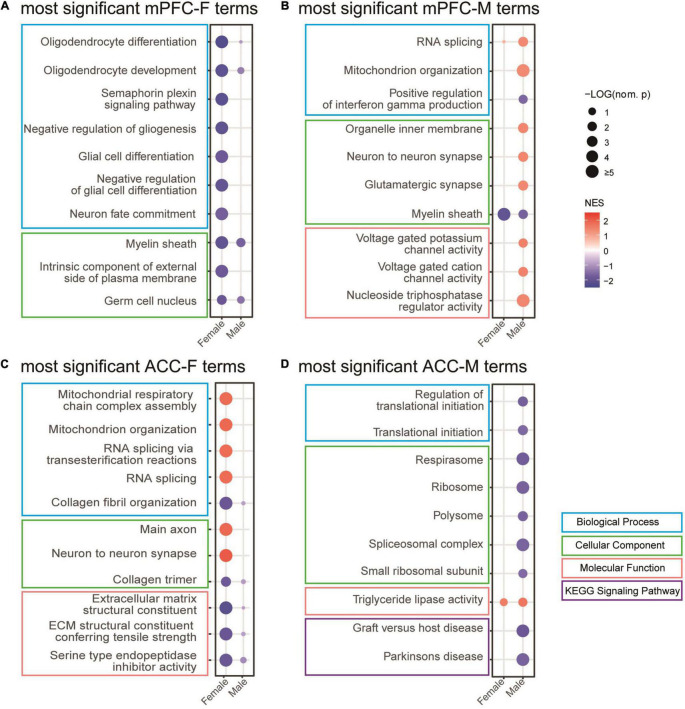
Gene set enrichment analysis (GSEA) of the transcriptomic changes in the mPFC and the ACC of female and male mice at 2 weeks after SNI. Most significantly regulated gene sets in the mPFC of female (mPFC-F, **A**) and male (mPFC-M, **B**) mice, as well as in the ACC of female (ACC-F, **C**) and male (ACC-M, **D**) mice, comparing the sham versus SNI groups. The size of the dots indicates the –log_10_ (nominal *p*) values, and the color indicates the normalized enrichment scores (NES).

We combined the GO and KEGG terms that were most significantly different between sham versus SNI groups to generate the list of top 10 gene sets for each condition (Figure 6). The results showed that for the female mPFC, the most significantly regulated gene sets included oligodendrocyte differentiation, semaphoring plexin signaling pathway, negative regulation of gliogenesis, neuron fate commitment, intrinsic component of external side of plasma membrane, and germ cell nucleus (Figure 6A), which were all de-enriched after SNI. For the male mPFC, the gene sets that emerged as the most significant changes by SNI included enrichment of RNA splicing, mitochondrion organization, glutamatergic synapse, voltage gated potassium channel activity, and nucleoside triphosphatase regulator activity; de-enrichment of positive regulation of interferon gamma production and myelin sheath (Figure 6B). Of note, most of the identified gene sets were significantly enriched in one sex, but showed no or minimal enrichment in the other sex. The exceptions were myelin sheath and germ cell nucleus in the SNI group, which showed similar degree of de-enrichment in the mPFC of both sexes (Figures 6A,B and Supplementary Table 3).

For the ACC, the GSEA results revealed that the most significant changes by SNI in the female included enrichment of mitochondrial respiratory chain complex assembly, RNA splicing *via* transesterification reactions, main axon and neuron to neuron synapse; and de-enrichment of collagen fibril organization, collagen trimer, extracellular matrix structural constituent, and serine type endopeptidase inhibitor activity (Figure 6C). In the male ACC, the top list included enrichment of triglyceride lipase activity; and de-enrichment of regulation of translational initiation, respirasome, ribosome, spliceosomal complex, graft versus host disease, and Parkinson’s disease (Figure 6D). Similar to the observation in the mPFC, the gene sets in the ACC also showed sex-divergent patterns (Figures 6C,D and Supplementary Table 3).

Taken together, these data suggest that the GO and signaling pathways of the transcriptomic changes of the mPFC and the ACC at 2 weeks after SNI are also largely sex-specific.

### The Transcriptomic Profiles Between the Medial Prefrontal Cortex and the Anterior Cingulate Cortex Are Partially Distinct and Have Sex-Specific Properties in the Non-spared Nerve Injury Condition

**FIGURE 7:**
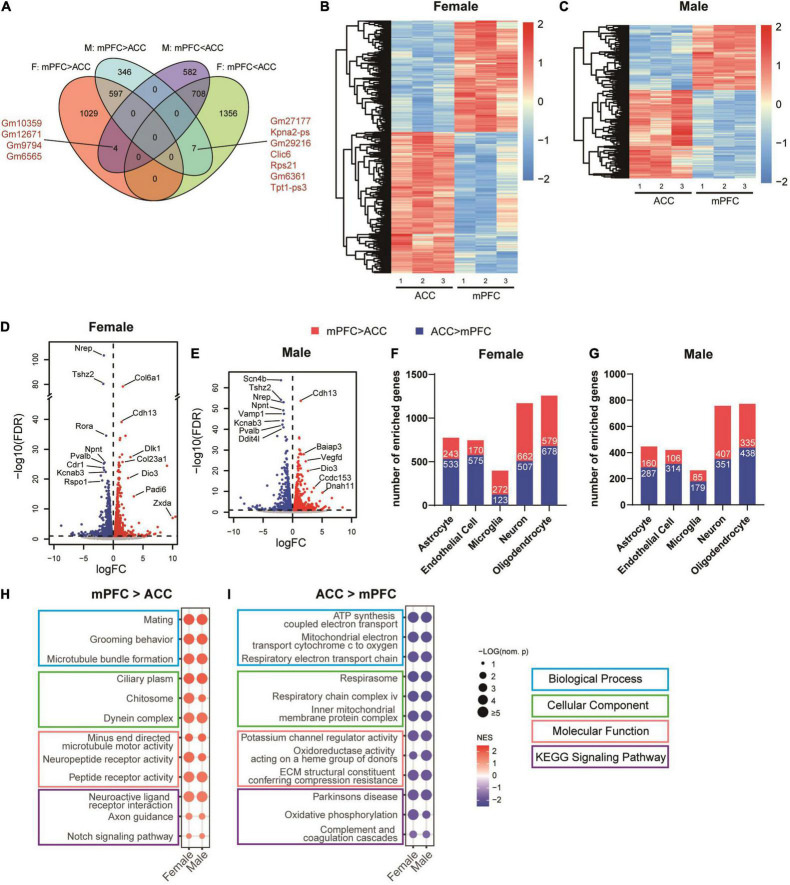
Differences between the transcriptomic profiles of the mPFC versus the ACC of female and male mice in the sham group. **(A)** Venn diagram depicting partial overlaps of the DEGs comparing the mPFC versus the ACC between the female and male mice. F, female; M, male. mPFC > ACC: transcripts expressed significantly more in the mPFC than in the ACC; mPFC < ACC: transcripts expressed significantly more in the ACC than in the mPFC. Hierarchical clustered heatmaps showing relative expression of the DEGs comparing the ACC versus the mPFC in the female **(B)** and male **(C)** mice from the sham group. Color scales represent standardized rlog-transformed values across samples. The volcano plots of the DEGs in the female **(D)** and male **(E)** mice, with horizontal lines at the –log_10_ (false discovery rate, FDR) = 1 and vertical lines at log_2_ (fold change, FC) = 0. Blue dots represent transcripts expressed significantly more in the ACC than the mPFC, and the red dots represent transcripts expressed significantly more in the mPFC than the ACC. The numbers of DEGs enriched in different cell types in the female **(F)** and male **(G)** mice, comparing the mPFC versus the ACC. Top differentially enriched gene sets in the mPFC **(H)** versus the ACC **(I)**. The size of the dots indicates the –log_10_ (nominal *p*) values, and the color indicates the normalized enrichment scores (NES).

We next wondered whether the brain area and sex-specific differences existed in the transcriptomic changes regulated by SNI in the sham (non-SNI) group of mice. Toward that end, we first compared the transcriptomic profiles between the mPFC and the ACC in the same sex of the sham groups. The DEG analyses identified 1630 transcripts in female mice and 950 transcripts in male mice that were significantly enriched in the mPFC, among which 597 were shared DEGs in both sexes. For the transcripts significantly enriched in the ACC, 2071 in female and 1294 in male mice were found, among which 708 DEGs were shared by both sexes (Figure 7A and Supplementary Table 4). Overall, more DEGs were identified in the female than the male mice, and similar amounts of DEGs were enriched in the mPFC versus the ACC (Figures 7B,C). Of note, between the mPFC and the ACC, *Dio3* (iodothyronine deiodinase 3) was among the most significantly enriched transcripts in the mPFC of both sexes (Figures 7D,E). It encodes an enzyme catalyzing the inactivation of thyroid hormone, and is involved in regulating aggressive and maternal behaviors (Stohn et al., 2016, 2018). The top DEGs enriched in the ACC and shared by both sexes included *Npnt* (nephronectin), a member of the epidermal growth factor-like superfamily; *Pvalb* (parvalbumin), the marker for fast-spiking inhibitory neurons regulating the excitability of the targeted neurons; and *Kcnab3* (potassium voltage-gated channel subfamily a regulatory beta subunit 3), a component of potassium channel (Figures 7D,E). The identified DEGs were enriched across multiple cell types in both sexes, with the highest enrichment in oligodendrocytes and neurons (Figures 7F,G). Further GSEA analyses revealed that the most significantly enriched gene sets in the mPFC of both sexes were related with mating, grooming behavior, microtubule formation, neuropeptide receptor activity, axon guidance and notch signaling pathway (Figure 7H). On the other hand, the top gene sets enriched in the ACC of both sexes were related with mitochondrial function, potassium channel regulator activity, extracellular matrix, Parkinson’s disease, oxidative phosphorylation as well as complement and coagulation cascades (Figure 7I and Supplementary Table 5). Of note, the gene sets of mitochondrial function, potassium channel regulator activity, extracellular matrix and Parkinson’s disease were also among the most significant ones regulated by SNI, indicating that the differences in transcriptomic changes after SNI in the mPFC versus the ACC are at least in part due to the distinct patterns of transcriptomes between these two regions at baseline.

**FIGURE 8:**
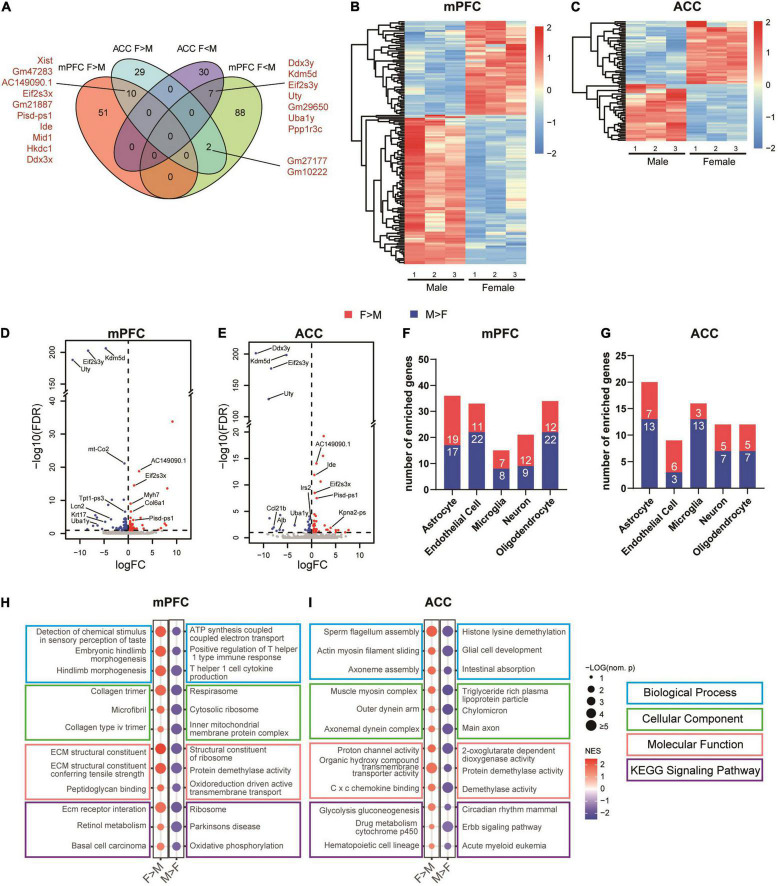
Sex differences in the transcriptomic profiles of the mPFC and the ACC in the sham group. **(A)** Venn diagram depicting partial overlaps of the sex-specific DEGs between the mPFC and the ACC. F > M: transcripts expressed significantly more in the female than in the male mice; F < M: transcripts expressed significantly more in the male than in the female mice. Hierarchical clustered heatmaps showing relative expression of the sex-specific DEGs in the mPFC **(B)** and the ACC **(C)** from the sham group. Color scales represent standardized rlog-transformed values across samples. The volcano plots of the sex-specific DEGs in the mPFC **(D)** and the ACC **(E)**, with horizontal lines at the –log_10_ (false discovery rate, FDR) = 1 and vertical lines at log_2_ (fold change, FC) = 0. Blue dots represent transcripts expressed significantly more in the male than in the female mice, and the red dots represent transcripts expressed significantly more in the female than in the male mice. The numbers of specific DEGs enriched in different cell types in the mPFC **(F)** and the ACC **(G)**. Top sex-specific differentially enriched gene sets in the mPFC **(H)** and the ACC **(I)**. The size of the dots indicates the –log_10_ (nominal *p*) values, and the color indicates the normalized enrichment scores (NES).

To further characterize sex-dependent transcriptomic signatures in the brain regions of interest, we performed DEG analyses of the female versus male transcriptomes in the mPFC and the ACC of the sham groups. The results showed that the expression levels of 61 transcripts of the mPFC and 41 transcripts of the ACC were higher in the female than the male mice. Ten of these transcripts were shared by the mPFC and the ACC, among which *Xist*, *Eif2s3x*, and *Ddx3x* are located within the X chromosome (Figure 8A). The expression levels of 97 transcripts in the mPFC and 37 transcripts in the ACC were significantly higher in the male than the female mice. Seven of these transcripts were shared by the mPFC and the ACC, among which *Ddx3y*, *Eif2s3y*, *Uty*, and *Uba1y* are located within the Y chromosome (Figure 8A and Supplementary Table 6). More DEGs between female and male mice were identified in the mPFC than the ACC. In the mPFC, more DEGs were enriched in the male than female mice, whereas similar amounts of DEGs were enriched in the female and male ACC (Figures 8B,C). Beyond the genes located in X or Y chromosomes, the noticeable top enriched transcripts in the female mPFC included *Myh7*, which encodes myosin heavy chain 7, and *Col6a1*, which encodes collagen type VI alpha 1 chain, the basic structural unit of collagen VI. The most significantly enriched transcripts in the male mPFC included *Tpt1-ps3* (tumor protein, translationally controlled, pseudogene 3) and *Krt17* (keratin 17), both of which regulate tumorigenesis (Fiucci et al., 2003; Yang et al., 2019; Figure 8D). The noteworthy sex-different transcripts in the ACC included *Ide* (insulin degrading enzyme; female > male), which regulates the catabolism of insulin and β-amyloid (Tundo et al., 2013); and *Irs2* (insulin receptor substrate 2, male > female), which provides an alternative route for insulin signaling (Copps and White, 2012; Figure 8E). The differential expression of *Ide* and *Irs2* indicates the possibility of distinct responses to insulin in the female versus male ACC. The identified DEGs were enriched across multiple cell types in the mPFC and the ACC (Figures 8F,G). We further performed GSEA analyses to compare female versus male mPFC and ACC. We found sex-differences across multiple domains of GO and KEGG signaling pathways in both brain regions. The sex-specific gene sets in the sham groups were partially overlapped with the most significantly altered gene sets in different sexes by SNI (Figures 6A–D, 8H,I and Supplementary Table 7).

Collectively, these findings suggest the already existed difference of transcriptomic profiles between the mPFC and the ACC of non-SNI sham group in a sex-specific manner, which may contribute to the region- and sex-specific alternations in the transcriptomic profiles induced by SNI.

## Discussion

In summary, the current study reveals the distinct nature of the transcriptomic profiles of the mPFC and the ACC in a sex-specific manner at 2 weeks after SNI, an early time point when mice began to show mild depression-like phenotypes. Given that sex difference exists in susceptibility to both pain and depression (Bartley and Fillingim, 2013; Altemus et al., 2014), and that the mPFC and the ACC are known brain regions for their critical but opposite roles in the comorbidity of pain and depression (Barthas et al., 2015; Sellmeijer et al., 2018; Kummer et al., 2020), our findings provide a valuable resource for elucidating the molecular mechanisms underlying the differential regulation of the mPFC and the ACC in different sexes during early development of the comorbidity of pain and depression. In addition, these findings should pave the way for the development of targeted therapeutics for the treatment of chronic pain and its affective disorders.

Several epidemiological studies of chronic pain have well documented that the prevalence of chronic pain and depression is higher in women than men (Williams et al., 2003; Fillingim et al., 2009; Ruau et al., 2012; Vetvik and MacGregor, 2017). Although social and cultural factors may contribute to the differences (Meints and Edwards, 2018; Mills et al., 2019), biological factors cannot be ignored. However, the preclinical studies of pain and its associated affective disorders have been largely relied on male rodents. Direct comparison of the similarity and differences between sexes have rarely been reported (Mogil, 2020). In the current study, we first compared the mechanical pain and depression-like behaviors in female and male mice after SNI surgery, which, however, did not exhibit significant sex differences, except that the decrease in mechanical pain threshold was relatively larger in the female mice than in the male mice. We reason that several factors may contribute to the lack of sex-differences in our behavioral tests. First, SNI is a procedure that generates robust and persistent stimulation to pain fibers, which is hard to tolerant (Decosterd and Woolf, 2000; Guida et al., 2020), whereas sex-differential sensitivity to pain may be more relevant to lower magnitudes of painful stimuli. Second, the sex differences in tolerance to chronic pain and development of associated depressive phenotypes are likely due to in part the differential adaptation to chronic pain conditions. The current study only examined behavioral changes over the first 4 weeks after SNI, but sex-contributed phenotypic difference may require more time to emerge. Third, we cannot exclude the possibility that the behavioral tests used in this study weren’t sensitive enough to reveal the sex differences.

Despite limited differences at the behavioral level were detected, robust sex differences were observed at the transcriptional level after SNI. Interestingly, comparing the sham group versus the SNI group, more DEGs were identified for the male mice than the female mice in the mPFC, whereas the opposite patterns occurred in the ACC, indicating differential modulation of the two brain regions in different sexes. Consistent with this notion, Jones and Sheets reported that SNI selectively increased the excitability and the excitatory synaptic currents of the parvalbumin-expressing (PV^+^) inhibitory neurons in the layer 5 of the mPFC of male mice but not female mice (Jones and Sheets, 2020). As PV^+^ neurons are critical for controlling the excitability of the targeted pyramidal neurons (Hu et al., 2014), the change mentioned above may lead to greater reduction of overall excitability in the mPFC of the male mice than female mice, which in turn is thought to contribute to greater attention deficits in male and female individuals with chronic pain (Shiers et al., 2018; Jones and Sheets, 2020). On the other hand, women but not men with chronic low back pain showed alterations in the functional connectivity of the subgenual ACC (sgACC) (Osborne et al., 2021). It would be of interest to examine how the sex-specific and distinct changes in the transcriptomic profiles of the mPFC and the ACC may be related to their functional alterations during neuropathic pain.

In addition to the differences in the numbers of DEGs, we found that the DEGs induced by SNI in the mPFC and the ACC showed little overlaps between female and male mice. The GSEA results further revealed largely distinct enrichment of transcriptomes in the GO and signaling pathways induced by SNI in female versus male mice. These findings are consistent with the emerging evidences, suggesting that clear differences existed in the mechanisms underlying chronic pain processing and the development of affective disorders during chronic pain progression at the system, cellular, and molecular levels (Barthas et al., 2015; Zhu et al., 2021). Such mechanistic differences may contribute to the differential sensitivity and tolerance of chronic pain and susceptibility to pain-induced depression, as well as differential treatment options for female and male patients (Bartley and Fillingim, 2013; Meints and Edwards, 2018). By directly comparing female and male transcriptomes in the two brain hub regions for the comorbidity of pain and depression, our study provides a useful resource for further dissecting and understanding the mechanisms contributing to the dynamic interaction of neuropathic pain and depression.

The sex-differences in the transcriptomic changes of the mPFC and the ACC after SNI were partially overlapped with the sex-differences in the transcriptomes of the two regions in the sham group. These findings suggest that the already existed biological differences in the female and male brains, which may be due to genetic, genomic imprinting, and hormonal differences in different sexes (Ngun et al., 2011; Mogil, 2012; Choleris et al., 2018), may contribute to their differential responses and adaption to chronic pain and its affective disorders like depression.

While the mPFC and the ACC are both activated by acute pain, chronic pain leads to opposite phenotypes in the two regions: hypoactive mPFC and hyperactive ACC accompanied by long-lasting changes in synaptic plasticity (Bliss et al., 2016; Sellmeijer et al., 2018; Tan and Kuner, 2021). However, direct comparison of the mechanisms underlying the divergent alterations of the mPFC versus the ACC by chronic pain remains under-investigated. Here, we showed that the transcriptomic profiles between the mPFC and the ACC were dramatically different in the sham condition and after SNI. Interestingly, after SNI, the most noticeable changes in the mPFC involved the downregulation of transcripts related to myelin formation, glia development, and axonal transportation. These processes are known to be prerequisite for the establishment and maintenance of proper functions of neural circuits during developmental stages and in the adulthood (Almeida and Lyons, 2017; Monje, 2018; Stadelmann et al., 2019). Therefore, we speculate that the impairment of these processes during early phase of neuropathic pain likely weakens the functional connectivity of the mPFC, which then contributes to the development of hypoactive phenotype. On the other hand, the most significantly upregulated transcripts in the ACC include *Syt6* and *Syt2*, two genes that encode different subtypes of synaptotagmins. Synaptotagmin-2 is one of three major synaptotagmins that are required for fast synchronous neurotransmitter release from presynaptic terminals, which also play important roles in long-term potentiation (LTP) and short-term synaptic plasticity of excitatory synapses (Chen et al., 2017; Wolfes and Dean, 2020). Synaptotagmin-6 is a key component of the acrosomal exocytosis process, which involves in exocytosis of neuropeptides such as BDNF (Dean et al., 2009). The upregulation of the *Syt6* and *Syt2* expression in the ACC after SNI may be involved in the regulation of LTP and hyperactivity of ACC neurons. However, it should be emphasized that increasing evidences have suggested that chronic pain induces distinct changes in diverse subtypes of neurons (including excitatory and different types of inhibitory neurons) in different layers of the mPFC and the ACC (Bliss et al., 2016; Tan and Kuner, 2021). Therefore, it is important to investigate in the next steps which neuronal populations are most affected by the key transcriptomic changes induced by SNI in the mPFC and the ACC.

How are changes at the transcriptional level in the mPFC at 2 weeks after SNI surgery related to the pathological development of depression during neuropathic pain? It has been reported previously that the PFC in patients with depression exhibits a hypoactive feature (Kupfer et al., 2012; Li et al., 2016). The PFC and the nucleus accumbens (NAc) receive dopaminergic innervation from the ventral tegmental area (VTA), also known as the brain’s reward circuits that are involved in the regulation of the susceptibility of depressive phenotypes, pain perception, and addictive behaviors (Russo and Nestler, 2013; Navratilova and Porreca, 2014). Optogenetic activation of glutaminergic projection of mPFC-to-NAc resulted in increased resilience to stress-induced depressive phenotypes (Bagot et al., 2015), while optogenetic suppression of VTA-to-mPFC neurons promoted susceptibility (Chaudhury et al., 2013), results that are in agreement with the numerous literatures on human and animal studies that support a general function of the mPFC to render individual resistance to stress and other negative stimuli, whereas dysfunction of mPFC can result in increased susceptibility to depression (Gomes and Grace, 2017; Hare and Duman, 2020). Importantly, elevation of the mPFC activity in the animal models, which also affect the reward circuits as mentioned above, can alleviate depression-like behaviors as well as hyperalgesia, suggesting that the mPFC acts as a hub which can regulate both pain and depression (Kummer et al., 2020; Liang et al., 2020). It is worth noting that in the clinical settings, repeated, non-invasive electrical or magnetic field stimulation targeting the mPFC has been found to ameliorate chronic pain as well as depressive symptoms, at least for a subset of patients who showed altered mPFC activity and connectivity (Borckardt et al., 2017; Ong et al., 2019; Hare and Duman, 2020; Scangos et al., 2021; Tan and Kuner, 2021). Ketamine, a fast-acting antidepressant, also appears to rapidly induce elevation of mPFC activity (Berman et al., 2000; Hare and Duman, 2020). These findings highlight the mPFC as an intercepted region for developing the comorbidity of pain and depression, and the importance of elucidating the molecular signatures and their underlying mechanisms that may contribute to the regulation of the neuronal activity of mPFC and its connecting strength to other brain regions in the reward circuits.

How does the mPFC become hypoactive in both chronic pain and depression? A growing number of studies have suggested the potential involvement of non-neuronal brain cells in depression. During the early pathogenesis of depression, alterations of non-neuronal brain cells may precede and lead to neuronal dysfunction (Cathomas et al., 2022). In line with this notion, we found in the current study that downregulation of myelination was the most striking change in the mPFC of both female and male mice at 2 weeks after SNI, when depressive-like behaviors began to emerge. In the mPFC of male mice after SNI, more than half of the downregulated transcripts were enriched in the oligodendrocytes. The myelin-related and oligodendrocyte-enriched genes, such as *Mal*, *Cnp*, *Plp1*, *Mobp*, *Mbp*, and *Mog* were among the most downregulated genes as well as the most strongly associated hub genes in the PPI network in the male mPFC. On the other hand, for the mPFC in female mice after SNI, we observed modest changes in the expression of individual genes enriched in oligodendrocytes and engaged in myelination, but highly coordinated expressional changes across multiple genes in the related gene sets. These led to the findings from our GSEA analysis that downregulation of gene sets involved in oligodendrocyte development and myelin sheath were the most prominent features after SNI. Thus, although gene expression patterns differ greatly between female and male mice after SNI, dysfunction of myelination process emerged to be the most pronounced alteration in the mPFC in both sexes. Interestingly, recent studies have also found decreases in oligodendrocytes and impairment of myelin in the mPFC after several types of stress that produce depressive phenotypes (Liu et al., 2012; Makinodan et al., 2012; Bonnefil et al., 2019). Selective demyelination of the mPFC induced by focal injection of lysolecithin reduced social interaction, a depression-related phenotype in mice, whereas clemastine, a compound that induced oligodendrocyte differentiation in the mPFC, alleviated depressive-like social avoidance induced by prolonged social isolation (Liu et al., 2016; Bonnefil et al., 2019). Oligodendrocytes in the mPFC have also been reported to play an important role in pain management. Downregulation of myelin-related proteins and oligodendrocyte apoptosis were observed in rats with fentanyl-induced hyperalgesia, whereas prophylactic blockage of oligodendrocyte apoptosis in the mPFC prevented hyperalgesia to occur (Wang et al., 2022). Together with these findings, our study suggests that restoring oligodendrocytes and myelin in the mPFC may be key to the treatment of comorbidity of pain and related depression.

In addition to the mPFC, the ACC has also been identified as a critical region in the pathological development of depression, particularly in the context of chronic pain (Barthas et al., 2015; Tan and Kuner, 2021). Abnormally increased activities in the sgACC and the perigenual ACC (pgACC) have been observed in patients with major depressive disorder and has been correlated with anhedonia, a typical symptom of depression (Philippi et al., 2015; Rupprechter et al., 2021; Pizzagalli and Roberts, 2022). The fast-acting antidepressant ketamine reduced sgACC and pgACC activity. Importantly, this reduction was associated with an anti-anhedonia effect at the behavioral level (Alexander et al., 2021). In neuropathic pain, lesion or inactivation of ACC reduced pain-related depressive symptoms, whereas activation of the ACC induced aversion to the place of its administration (Barthas et al., 2015; Zhang et al., 2017). Therefore, the ACC is also a key brain region at the interface between pain and depression.

In the present study, the transcriptome of ACC was significantly altered by SNI in the female mice, while only a limited numbers of DEGs were detected in the ACC of male mice. Given the importance of ACC in depression (Etkin et al., 2011; Pizzagalli and Roberts, 2022), and female subjects being more prone to depression than male subjects (Altemus et al., 2014; Bangasser and Cuarenta, 2021), we speculate that the sex-differences in transcriptional changes in the ACC after SNI may contribute to the differential prevalence of depression as neuropathic pain progresses. This speculation, however, awaits further experimental testing. In the ACC of female mice after SNI, besides upregulation of the synaptotagmins, the most notable change was the reduction of genes encoding extracellular matrix components. These genes also constituted the central sub-network of hub genes in the PPI network of the DEGs in the female ACC after SNI. Furthermore, GSEA analysis of the ACC transcriptome of female mice showed that downregulation of collagen fibril organization, collagen trimer, extracellular matrix structural constituent, and serine type endopeptidase inhibitor activity were the most prominent changes after SNI. Collectively, these findings reveal that the disruption of extracellular matrix in the ACC of female mice is a major event accompanying the early development of depressive-like phenotypes at 2 weeks after SNI surgery.

Extracellular matrix, which accounts for 20% of the brain’s volume, not only provides a supporting scaffold for other brain cells, but also acts as the first messengers for transmission of extracellular signals to modulate neuronal functions (Dityatev et al., 2010; Barros et al., 2011; Laham and Gould, 2021). Formation of extracellular matrix at the end of the “critical period” during visual cortex development serves as an essential mechanism to restrain structural plasticity (Hensch, 2005; de Vivo et al., 2013). In the adulthood, it has been reported that remodeling of extracellular matrix is required for *de novo* synapse formation, various forms of synaptic plasticity, and fear memory erasure (Tajerian et al., 2018; Laham and Gould, 2021). In the context of neuropathic pain, Kawasaki et al. (2008) reported the distinct roles of acutely up-regulated matrix metalloproteases-9 and delayed induction of matrix metalloproteases-2, two proteases involved in the breakdown of extracellular matrix and cytokines, in the dorsal root ganglion for the development of early- and late-phase neuropathic pain indued by spinal nerve ligation. Inhibition of matrix metalloproteases-9 and matrix metalloproteases-2 by pharmacological or siRNA-based strategies effectively produced anti-allodynic effect (Kawasaki et al., 2008). Li et al. (2021) reported that SNI reduced the expression of LAMB1, a major component of extracellular matrix in the ACC. Knockdown of LAMB1 in the ACC increased the release probability of neurotransmitters and led to abnormal postsynaptic spine remodeling, which in turn increased pain sensitivity and caused depression-like behaviors (Li et al., 2021). Our findings now showed that the decrease of extracellular matrix is a major event in the early development of depressive phenotypes after SNI, further highlight the restoration of extracellular matrix in the ACC as a potential therapeutic strategy for the treatment of pain and depression comorbidity.

In conclusion, our study reveals that at 2 weeks after SNI, an early time point when the mice began to show mild depressive symptoms, the transcriptomic changes in the mPFC and the ACC are highly distinct and sex-specific. Female mice exhibited stronger transcriptomic changes in the ACC than male mice, while the opposite was observed in the mPFC. The transcriptomic changes occurred across multiple brain cell types. We further identified downregulation of myelin-related transcripts in the mPFC of both sexes, as well as upregulation of synaptotagmins and downregulation of extracellular matrix components in the female ACC as the most prominent changes induced by SNI. Taken together, these findings demonstrate the transcriptional dimorphism in both sexes and brain areas induced by neuropathic pain, suggesting potential therapeutic targets for the treatment of chronic pain and its related affective disorders.

## Data Availability Statement

The datasets presented in this study can be found in online repositories. The names of the repository/repositories and accession number(s) can be found below: https://www.ncbi.nlm.nih.gov/geo/, GSE197233.

## Ethics Statement

The animal study was reviewed and approved by the Institutional Animal Care and Use Committee of Sun Yat-sen University.

## Author Contributions

XY and W-JL designed the study. WD, SH, XC, and YZ carried out the RNAseq analyses. WD, YL, PZ, PX, and MY carried out the experiments. XY, W-JL, SH, and WD wrote the manuscript. All authors contributed to the article and approved the submitted version.

## Conflict of Interest

The authors declare that the research was conducted in the absence of any commercial or financial relationships that could be construed as a potential conflict of interest.

## Publisher’s Note

All claims expressed in this article are solely those of the authors and do not necessarily represent those of their affiliated organizations, or those of the publisher, the editors and the reviewers. Any product that may be evaluated in this article, or claim that may be made by its manufacturer, is not guaranteed or endorsed by the publisher.

## Footnotes

1
https://cloud.biohpc.swmed.edu/index.php/s/grcm38/download

2
https://pga.mgh.harvard.edu/primerbank/

3
https://www.ncbi.nlm.nih.gov/tools/primer-blast/index.cgi

4
https://blast.ncbi.nlm.nih.gov/Blast.cgi

## Supplementary Material

Supplementary Table 1 List of primers used in the qPCR validation of RNAseq results.

Supplementary Table 2 List of the DEGs in the mPFC and the ACC of female and male mice, comparing the sham versus the SNI groups.

Supplementary Table 3 Gene set enrichment analysis results for the mPFC and the ACC of female and male mice, comparing the sham versus the SNI groups.

Supplementary Table 4 List of the DEGs comparing the mPFC versus the ACC in the female and male mice of the sham groups.

Supplementary Table 5 Gene set enrichment analysis results for the female and male mice, comparing the mPFC versus the ACC in the sham groups.

Supplementary Table 6 List of the DEGs in the mPFC and the ACC, comparing female and male mice of the sham groups.

Supplementary Table 7 Gene set enrichment analysis results for the mPFC and the ACC, comparing female and male mice of the sham groups.
