# Supplementary material for: Sex-Specific Transcriptomic Signatures in Brain Regions Critical for Neuropathic Pain-Induced Depression
Source: Front Mol Neurosci. 2022 May 18;15:886916. doi: 10.3389/fnmol.2022.886916 (PMC9159910; doi:10.3389/fnmol.2022.886916)
Supplement: Supplementary Table 1 — List of primers used in the qPCR validation of RNAseq results. [file Table_1.DOCX]

**Table 1 Primer sequences used for RT-PCR**

| Gene | Forward primer | Reverse primer |
| --- | --- | --- |
| *Gdf1* | CATGCCACGTGGAGGAACTA | CTGGAGGACAGACCGCTG |
| *Snhg14* | CCGTGTCAGTATGTCTGCTG | CATGAGTTGGGAACGCCATT |
| *Myh14* | CAACCTGCGAGAACGCTACTA | CTTGCCCCGGTACATTTCAAC |
| *Tmem267* | ACAGAATCCCTCATTTCCAGCA | CCGATCACTCCGTGTACTATGT |
| *Trf* | GCTGTCCCTGACAAAACGGT | CGGAAGGACGGTCTTCATGTG |
| *Gfap* | CGGAGACGCATCACCTCTG | AGGGAGTGGAGGAGTCATTCG |
| *Mbp* | GCAGAGCTTCCGACTATAAAT | TCTCTTCCTCCCAGCTTAAA |
| *Cnp* | TTTACCCGCAAAAGCCACACA | CACCGTGTCCTCATCTTGAAG |
| *Scn4b* | ACCATCTACGCTATTAACGGCT | CGCTGTTATTGTAGGACCACTT |
| *Syt2* | AGAACCTGGGCAAATTGCAGT | CCTAACTCCTGGTATGGCACC |
| *Acta2* | GTCCCAGACATCAGGGAGTAA | TCGGATACTTCAGCGTCAGGA |
| *Col1a1* | GCTCCTCTTAGGGGCCACT | CCACGTCTCACCATTGGGG |
| *GM27177* | CGCAGGGGCATTCATGATTG | CCCCAGCTCAAGGCCATTTA |
| *Gm5641* | ACCGAAGAGAGCTGTTTCTA | ACTCTGCCTGTCTTCCATAA |
| *Gcat* | GGACAGCGAACTGGAAGGG | AGTTATTGGCACAGAAGTTGAGG |
